# Cortical auditory evoked potential in assessment of neonates: a study about minimum level of responses in term and preterm newborns^☆^

**DOI:** 10.1016/j.bjorl.2019.04.009

**Published:** 2019-07-04

**Authors:** Dayane Domeneghini Didoné, Lilian Sanches Oliveira, Alessandra Spada Durante, Kátia de Almeida, Michele Vargas Garcia, Rudimar dos Santos Riesgo, Pricila Sleifer

**Affiliations:** a Universidade Federal do Rio Grande do Sul (UFRGS), Programa de Pós-Graduação em Saúde da Criança e do Adolescente, Porto Alegre, RS, Brazil; b Santa Casa de São Paulo, Faculdade de Ciências Médicas, São Paulo, SP, Brazil; c Universidade Federal de Santa Maria, Porto Alegre, RS, Brazil; d Universidade Federal do Rio Grande do Sul, Departamento de Saúde e Comunicação Humana, Porto Alegre, RS, Brazil

**Keywords:** Infant, newborn, Electrophysiology, Auditory threshold, Evoked potentials, auditory, Recém-nascido, Eletrofisiologia, Limiar auditivo, Potenciais evocados auditivos

## Abstract

**Introduction:**

The study of the threshold level of cortical auditory response in adults has been investigated in previous studies. Due to maturational issues, little is known about these responses in neonates. Technological advances with automatic analysis devices now allow investigation in specific populations. Thus, new studies are needed to establish the feasibility of using this auditory potential to identify the lowest levels of responses in children.

**Objective:**

Verify and compare latency and amplitude in 80 dBnNA and the minimum level of cortical auditory response in term and preterm neonates.

**Methods:**

A cross-sectional, comparative study involving 59 neonates, 35 full-term births and 24 preterm births, with positive results in the Neonatal Hearing Screening. The Hearlab system was used to investigate the P1i auditory potential with tone burst stimulus at frequencies of 500, 1000, 2000 and 4000 Hz. The minimum response level search ranged from 80 to 0 dBNA and was detected automatically. The results were compared between groups, evaluating the latency and amplitude in 80 dBNA and the minimum level of cortical auditory response.

**Results:**

The mean values obtained for the minimum level of cortical auditory response in term group were 26 ± 8.81; 26.14 ± 6.97; 29 ± 7.65 and 29.43 ± 7.04 dBNA and for preterm neonates of 31.96 ± 10.41; 34.13 ± 11.34; 33.64 ± 11.03 and 37.73 ± 11.92 dBNA, for the frequencies of 500, 1000, 2000 and 4000 Hz, respectively. There was a difference between groups for the latency of P1i at 4000 Hz and the minimum response levels at 500, 1000 and 4000 Hz, with higher values for preterm infants.

**Conclusion:**

It was possible to obtain latency and amplitude values at 80 dBnNA and the minimum level of cortical response in term and preterm newborns, with different results between groups, with higher values in those born preterm.

## Resumo

**Introdução:**

A investigação do nível mínimo de resposta auditiva cortical tem sido alvo de diferentes estudos em adultos. Devido a questões de maturação, pouco se sabe sobre essas respostas em recém-nascidos. Com o avanço tecnológico, dispositivos de análise automática surgiram com o objetivo de retomar essa avaliação em populações específicas. Assim, novos estudos são necessários para verificar a viabilidade do uso desse potencial auditivo na obtenção de níveis mínimos de respostas na criança.

**Objetivo:**

Verificar e comparar latência e amplitude em 80 dBnNA e o nível mínimo de resposta auditiva cortical em recém-nascidos a termo e pré-termo.

**Método:**

Estudo transversal, comparativo, envolvendo 59 neonatos, 35 nascidos a termo e 24 pré-termos, com resultados positivos na triagem auditiva neonatal. O sistema *Hearlab* foi utilizado para investigar o potencial auditivo P1i com estímulo *tone burst* nas frequências de 500, 1000, 2000 e 4000 Hz. A busca do nível mínimo de resposta variou de 80 a 0 dBNA e foi detectado automaticamente. Os resultados foram comparados entre os grupos, avaliando a latência e amplitude em 80 dBNA e o nível mínimo de resposta auditiva cortical.

**Resultados:**

Os valores médios obtidos para o nível mínimo de resposta auditiva cortical no grupo nascido a termo foram 26 ± 8,81; 26,14 ± 6,97; 29 ± 7,65 e 29,43 ± 7,04 dBNA e para recém-nascidos Pré-termos foram de 31,96 ± 10,41; 34,13 ± 11,34; 33,64 ± 11,03 e 37,73 ± 11,92 dBNA, para as frequências de 500, 1000, 2000 e 4000 Hz, respectivamente. Houve diferença entre os grupos para a latência de P1i em 4000 Hz e os níveis mínimos de resposta em 500, 1000 e 4000 Hz, com valores maiores em Pré-termos.

**Conclusão:**

Foi possível obter valores de latência e amplitude em 80 dBnNA e o nível mínimo de resposta cortical em recém-nascidos a termo e Pré-termos, com resultados diferentes entre os grupos, com valores maiores em pré-termos.

## Introduction

Cortical auditory evoked potentials (CAEP) were discovered and used in electrophysiological threshold research since the 1960s and 1970s. Due to some technical difficulties and the discovery of brainstem auditory evoked potentials, this evaluation technique was abandoned by several researchers and even today is not used in clinical practice for this purpose, since it still lacks appropriate studies.

Some researchers believe that the use of CAEP has advantages over short-latency auditory evoked potentials, such as providing information about central auditory functions,^1^, ^2^ greater range of responses due to proximity of the electrodes with the generating source and better correlation with behavioral auditory thresholds.^3^, ^4^ In addition, in specific populations, such as patients with auditory neuropathy, electrophysiological threshold using CAEP would be the only reliable electrophysiological procedure to obtain the answers, because short-term potentials require neural synchrony.^5^, ^6^

Maturation of the CAEP occurs in late adolescence and the electrophysiological tracing is composed of the P1–N1–P2 complex. Visualization of this complex at different intensities makes it possible to obtain the cortical electrophysiological thresholds in this age group.^1^, ^2^, ^6^

In children, the immaturity of the central nervous system results in a different morphology of the CAEP than that seen in adults, with variations in latency and amplitude throughout development.^7^, ^8^ The morphology consists predominantly of a positive peak and a deflection,^9^ with latencies ranging from 200–250 ms for the positive peak and 350–450 ms for the deflection.^10^ Studies carried out in the infant population show that CAEP can be used to validate the effectiveness of hearing aids and cochlear implants^11^, ^12^, ^13^, ^14^ and in the maturational evaluation of the central nervous system of specific populations.^15^, ^16^ The assessment of cortical auditory responses thresholds in children during central auditory maturation is still poorly studied.^3^, ^4^ On the other hand, investigators have studied the CAEP at different intensity levels in children including infants^17^ and neonates to better understand its applicability.^5^, ^18^ Its use in young children is advantageous, since it is possible to obtain data in neonates who are either alert or during light sleep.^9^, ^17^, ^18^ Authors^19^ add that the use of CAEP in estimating the auditory threshold in young children who are difficult to test behaviorally or who can not be sedated could be possible as part of the audiological evaluation, but this has been explored very little in the scientific literature.

The development of automatic analysis equipment of CAEP allowed the clinician to rethink the use of this evaluation, including obtaining the threshold levels of cortical auditory response in different age groups. Researchers report that subjective analysis is still the most commonly used method, but may be vulnerable to errors of interpretation.^3^, ^12^ The HearLab System is an automatic analysis device developed by the National Acoustic Laboratories in Australia. It has high sensitivity for detecting cortical responses and reducing noise and artifacts; in the past responses had been more unstable and influenced by noise when compared to short-latency auditory evoked potentials.^20^

The detection sensitivity of the cortical auditory responses of the Hearlab System device is high compared with experienced examiners. Authors^20^ verified the effectiveness of the Hearlab System automatic response with experienced CAEP examiners and concluded that both the automatic analysis device and the examiners had high sensitivity in the detection of responses.

Among the clinical applications of the Hearlab System, we highlight the research of the minimum cortical response level by the Cortical Tone Evaluation (CTE) module. In a recent study, authors^2^ reported average electrophysiological responses in adults with normal hearing at 18.23 dBHL for 500 Hz, 15.9 dBHL for 1000 Hz, 15.97 dBHL for 2000 Hz and 17 dBHL for 4000 Hz. Other investigators^18^ obtained averages of electrophysiological responses in neonates of 24.8 dBHL at 500 Hz, 25 dBHL at 1000 Hz, 28 dBHL at 2000 Hz and 29.4 dBHL at 4000 Hz, exhibiting higher cortical auditory response thresholds compared to those in adults^2^ due to maturational issues. Other researchers^17^ reported that it is possible to obtain responses in infants, even at low intensities, showing the usefulness of CAEP for several clinical applications.

Because maturational issues influence auditory evoked potentials, CAEP assessment is required in different age groups and populations, such as preterm births. Thus, the automatic analysis of the responses has contributed to the better understanding and reliability of the same when obtained in children younger than 3 months,^18^ a crucial period for the early audiologic diagnosis. In addition, CAEP would be ideal for research on the minimal level of responses in cases of auditory neuropathy and neurological disorders.^5^

Therefore, due to the paucity of studies and the need for a better understanding of the possibility of obtaining the CAEP minimum level response in neonatal population of term and preterm births that may be influenced by maturational issues, the present research is undertaken.

Based on the above, the purpose of this study was to verify and compare the latency and amplitude in 80 dBnNA and minimum level of cortical response of term and preterm neonates.

## Methods

It is a cross-sectional and observational study, carried out by Universidade Federal do Rio Grande do Sul in partnership with Faculdade de Ciências Médicas da Santa Casa de São Paulo. This study was approved by the Research Ethics Committees of both Universities under protocols 44965015.8.1001.5334 and 51349315.6.1001.5479.

Only newborns participated in the study. Their parents or guardians, after receiving information about the objectives and methodology of the research, agreed to the procedures to be performed and signed the Free and Informed Consent Term.

The subject sample consisted of full-term and preterm babies from the outpatient clinic and the Neonatal Intensive Care Unit (NICU) of Faculdade de Ciências Médicas da Santa Casa de São Paulo.

In the maternity hospital, the parents or guardians were approached and invited to participate in the research. When they agreed, the evaluation date was provided by the researcher. In addition, the participants’ charts were analyzed with the purpose of verifying the inclusion and exclusion criteria of the present study. For the participants of the NICU, the subjects’ selection was performed, initially, through the verification of the medical records, also with the purpose of verifying the inclusion and exclusion criteria. After this stage, the parents or guardians were contacted, informed about the research, and, if there was interest and consent, the evaluation date was provided.

Initially, 114 subjects were contacted and 48 did not agree to participate. During the first month, 66 neonates were evaluated. After analyzing the results, 7 subjects were excluded due to the inconclusive results due to the lack of cooperation of the neonates and consequently, excessive noise level during the evaluation. The final sample consisted of 59 subjects, 35 full term (16 female and 19 male) and 24 pre-term (11 female and 13 male). Newborns were considered preterm if they had gestational age less than or equal to 36 weeks.^21^ The subjects were of both sexes and had positive results in Neonatal Hearing Screening in both ears. Neonates were assessment through Transient-Evoked Otoacoustic Emissions (TEOAEs) and/or automated brainstem auditory-evoked response, performed prior to hospital discharge, according to the recommendations of the national^22^ and international committees^23^ for the presence or absence of risk indicators for hearing loss. Neonates without risk indicators were submitted to TOAEs and neonates with risk indicators for TOAEs and automated brainstem auditory-evoked response.

The TEOAEs and/or automated brainstem auditory-evoked responses were examined using a Madsen Accuscreen device (GN Otometrics, Kastrup, Denmark). For automated brainstem auditory-evoked responses, the electrodes were fixed at the vertex (active), at the position of the C7 vertebra (reference) and at the zygomatic. Results obtained at 35 dBHL with click stimuli were considered satisfactory.^24^ A satisfactory/positive TEOAE result was considered to be a signal-to-noise ratio greater than or equal to 3 dB for a frequency of 1000 Hz and 6 dB for frequencies above 1000 Hz (at least 3 of the 5 frequencies surveyed) bilaterally.^25^

The following inclusion criteria were used for full-term infants: gestational age greater than or equal to 37 weeks, absence of risk indicators for hearing loss,^22^, ^23^ in good health, a positive result in Neonatal Hearing Screening test and electrophysiological record of adequate morphological quality. For preterm group, the following inclusion criteria were adopted: gestational age less than or equal to 36 weeks, in good health, a positive result in Neonatal Hearing Screening test and electrophysiological record of adequate morphological quality. Subjects with neurological abnormalities, syndromes and those who did not cooperate during the exam were excluded from the study. Subjects with hearing impairment syndromes, family history of hearing impairment, congenital anomalies, neurological disorders, congenital infection, bacterial meningitis, and blood transfusion were also excluded from the preterm and term group.^26^

In this study, 10 preterm did not present risk indicators for hearing loss and 14 presented additional risk indicators, such as NICU stay for more than five days, ototoxic use, birth weight less than 1500 g and need for ventilation mechanics. A previous statistical analysis was performed and did not show differences between the groups (preterm with and without risk indicators) for all the variables of this study (latency, amplitude and minimum level of response in all acoustic stimuli tested) (analysis of variance, ANOVA, *p* > 0.05). Thus, all subjects were considered in a single group (preterm group).

The subjects in term and preterm groups were evaluated between 38 and 43 weeks postconceptional age, being the corrected age for preterm births.

In the present research the cortical potential P1 was analyzed, characterized by a peak between 150 and 400 ms, which was designated P1i (P = Positive, 1 = first peak and I = infant). Such nomenclature was used in order to differentiate the P1 described in the tracing of adult individuals.^27^ It should be noted that due to the type of stimulus used (tone burst), only a positive peak was observed in the electrophysiological tracing, reflecting the characteristics of the acoustic stimulus.

For the research of the P1i, Hearlab System was used. This equipment was developed in Australia by the National Acoustic Laboratories. The cortical tone evaluation module was used for assessment of CAEP in the frequencies of 500, 1000, 2000 and 4000 Hz at 80 dBHL. The minimum level of cortical auditory response was also assessment in these frequencies, in a monoaural way, being the choice of the aleatory side. The chosen ear side was made according to the position of neonate mother's lap. Studies^2^, ^18^, ^28^ do not show hemispheric differences for cortical auditory potentials.

The CAEP was examined by air conduction, with inserted ER-3A headphones. The equipment has previously been calibrated in dBHL, according to the technical specifications, by a qualified professional. It should be emphasized that for this study, the minimum level of cortical auditory bone response was not investigated, since it was not the main objective and due to the delay in the procedure, since the minimum response level was investigated in four frequencies. However, additional research is being done in the same lab.

The procedure was performed in an acoustically and electrically treated room. The parents or guardians sat in a comfortable chair, with the participants comfortably positioned on their laps. The temperature of the evaluation environment was controlled and maintained at 24 °C and the ambient noise did not exceed 35 Db (A).^2^

The electrodes were fixed in the following positions: active electrode in vertex (Cz) (middle line of head), the ground electrode at the forehead (Fpz), and the reference electrodes on the right or left Mastoid (M2 or M1) after cleansing the skin with Nuprep abrasive paste. Their impedance did not exceed 5 kohms.^2^

In order to perform the electrophysiological research, neonates remained in light sleep or awake with minimal body movements, similar to other studies.^9^, ^18^ The Brazelton^29^ scale was used to identify the behavioral status of the newborns. This scale has six behavioral states: State 1: deep sleep; State 2: light sleep, closed eyes, some body movement; State 3: sleepy, opening and closing eyes; State 4: awake, eyes open, minimal body movements; State 5: fully awake, vigorous body movements; State 6: crying. Only neonates in Stages 2, 3 and 4 were included in the study.

The parameters for the research were tone burst stimuli, alternating polarity, cosine envelope, 1.125 ms stimulus interval, duration of 40 ms, velocity of 0.5 Hz, 10 ms rise-fall, 30 ms plateau, high pass filter of 0.16 Hz and low pass of 0.30 Hz, according to the equipment manual.^30^

The noise level was monitored throughout the evaluation. The maximum level of stimulus rejection considered was 20% of total stimuli. The equipment has a favorable control of artifacts and allows the audiologist to be monitored. Residual noise values less than or equal to 3.2 μV indicate good signal quality. Values between 3.2 and 3.6 μV were intermediate values and values greater than 3.6 μV indicate poor signal recording quality.^30^ In this study, the maximum value allowed for noise was 3.6 μV and, for this reason, two participants with extreme agitation and excessive movement were excluded.

The presence or absence of P1i cortical potential was automatically detected by the equipment, which applied Hotelling's T2 test to analyze the signal-to-noise ratio of the responses obtained at each frequency and intensity. In this case, each sample was divided into nine portions within the analysis period of 50 ms each in a window of up to 500 ms. The mean of each point was tested using the multivariate ANOVA. The applied statistical test determined whether the waveform hypothesis was different from random noise. Responses were considered present when the *p*-value was ≤5%; that is, when the response was greater than the noise and with at least 50 stimuli for each frequency tested. It should be emphasized that cortical auditory responses require fewer stimuli to be used due to the habituation of the central nervous system.^4^ When the response was considered present by the equipment with at least 50 stimuli, the evaluation was interrupted by the evaluator.

The tracings were not replicated due to the fact that the equipment has automatic analysis and does not allow the visualization of two tracings simultaneously. This protocol is in accordance with the recommendations^4^ for this type of procedure with automatic analysis equipment.

The latency and amplitude parameters at 80 dBHL of each wave for each frequency that was evaluated in this study were manually considered by three judges with experience in electrophysiology, because this marking was not performed by the equipment. The evaluations were performed blindly, without influence of the marking of the results according to the group. The examiners were instructed to perform the P1i marking on the highest positive peak observable within the 500 ms window. The amplitude was considered from the baseline (zero point) to the point of greatest amplitude of the wave.

**Figure 1:**
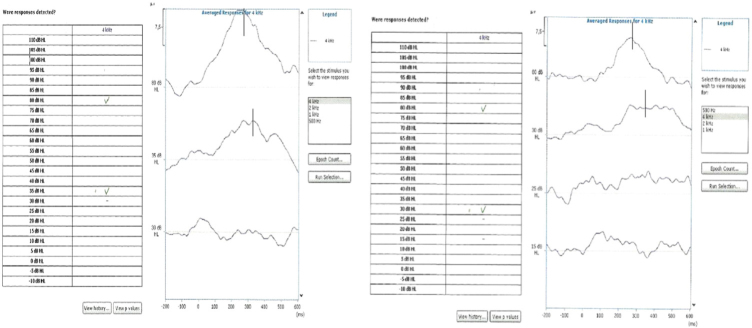
Example of minimum level cortical auditory response in subjects of the present research. The vertical line identifies the marking of the P1i component performed by the judges. In the first example the minimum level of cortical response obtained was 35 dBHL whereas in the second example it was 30 dBHL.

The assessment of minimum level of cortical auditory response started at an intensity of 80 dBHL. Soon after the intensity was decreased to 30 dBHL. In the absence of response, the intensity was increased in increments of 5 dBHL until reaching the threshold level of cortical auditory response. If the response was present at 30 dBHL, the stimulus intensity was decreased to 15 dBHL, shortly afterwards for 5 dBHL and for 0 dBHL. In the absence of response, the intensity was increased in increments of 5 dBHL, up to the minimum detection level of the cortical auditory response. The minimum level of cortical auditory response was determined by the presence or absence of a response verified automatically by the equipment. This protocol was proposed by one study,^31^ with small modifications described in another study,^18^ in order to facilitate the identification of responses. Fig. 1 illustrates the research on cortical auditory potential in 80 dBHL and the minimum level of response in research subjects.

The variables of latency, amplitude in 80 dBHL and the minimum level of auditory cortical response were compared between groups, and the data were tabulated in Excel spreadsheets and analyzed through the Statistical Package for Social Sciences (SPSS) version 20.0. The following statistical tests were used: Student's *t*-test, *U*-Mann–Whitney test and Chi-Square test for comparisons between groups. In addition, additional analyses such as Analysis of Variance (ANOVA) were performed. The significance level of 5% was adopted for all analyses.

## Results

Table 1 presents the descriptive data of the sample studied. Regarding the latency values of the cortical component P1i at 80 dBHL, a statistically significant difference was observed for the frequency of 4000 Hz, which was higher for the preterm neonates (Table 2). However, there were no statistically significant differences for the values of cortical potential amplitudes between groups (Table 3).

**Table 1:** Descriptive data of sample.

Variables	Total sample (*n* = 59)	Term (*n* = 35)	Preterm (*n* = 24)	*p*-value
Gestational age (weeks)	Average	39.52	34.05	<0.001^a^
	Minimum	37	28	
	Maximum	39.28	36	
	SD	1.16	2.39	
Age at the time of evaluation (weeks)	Average	40.91	40.37	0.11^a^
	Minimum	39	38	
	Maximum	43	43	
	SD	1.2	1.3	
Ear	Right	14	12	0.44^b^
	Left	21	12	
Sex	Female	16	11	0.99^b^
	Male	19	13	
Time of assessment (minutes)		70.94	65.54	0.20^a^

SD, Standard Deviation.

a Student's *t*-test for independent sample.

b Chi-Square test. The significance level of 5% was adopted.

**Table 2:** Latency values at 80 dBHL for the different frequencies between groups.

Frequency	Group	Average ± SD	Median	1°Q	2°Q	3°Q	Minimum	Maximum	*p*-value
500 Hz	Term (*n* = 35)	242.43 ± 39.88	244.00	209.00	244.00	277.00	168.00	313.00	0.92^a^
	Preterm (*n* = 23)	241.35 ± 48.79	237.00	202.00	237.00	267.00	169.00	371.00	
1000 Hz	Term (*n* = 35)	228.94 ± 34.98	229.00	208.00	229.00	253.00	157.00	307.00	0.25^a^
	Preterm (*n* = 23)	246.43 ± 47.49	237.50	208.00	235.00	258.00	200.00	370.00	
2000 Hz	Term (*n* = 35)	233.14 ± 42.65	236.00	205.00	236.00	256.00	137.00	353.00	0.97^a^
	Preterm (*n* = 22)	232.77 ± 36.54	231.50	201.50	231.50	256.00	170.00	310.00	
4000 Hz	Term (*n* = 35)	249.97 ± 49.16	243.00	213.00	243.00	271.00	157.00	370.00	0.05^a^
	Preterm (*n* = 23)	279 ± 60.49	277.00	234.00	269.00	302.00	178.00	398.00	

SD, Standard Deviation; Q, Quartile.

The significance level of 5% was adopted.

a Student's *t*-test for independent sample.

**Table 3:** Amplitude values at 80 dBHL for the different frequencies between groups.

Frequency	Group	Average ± SD	Median	1°Q	2°Q	3°Q	Minimum	Maximum	*p*-value
500 Hz	Term (*n* = 35)	6.71 ± 3.58	6.00	4.00	6.00	9.00	1.00	19.00	0.45^a^
	Preterm (*n* = 23)	7.72 ± 4.27	6.79	4.60	6.71	10.10	2.38	20.02	
1000 Hz	Term (*n* = 35)	7.08 ± 4.18	7.00	4.00	7.00	8.00	2.00	22.00	0.78^a^
	Preterm (*n* = 23)	7.73 ± 4.23	7.62	4.58	7.47	10.34	1.94	19.44	
2000 Hz	Term (*n* = 35)	5.85 ± 2.86	5.00	4.00	5.00	8.00	2.00	14.00	0.09^a^
	Preterm (*n* = 22)	7.31 ± 3.27	6.03	4.22	6.35	9.35	2.50	13.73	
4000 Hz	Term (*n* = 35)	5.82 ± 2.96	6.00	3.00	6.00	7.00	2.00	16.00	0.58^a^
	Preterm (*n* = 23)	6.87 ± 4.41	6.10	3.58	5.59	9.66	2.32	20.47	

SD, Standard Deviation; Q, Quartile.

The significance level of 5% was adopted.

a *U*-Mann–Whitney test.

**Figure 2:**
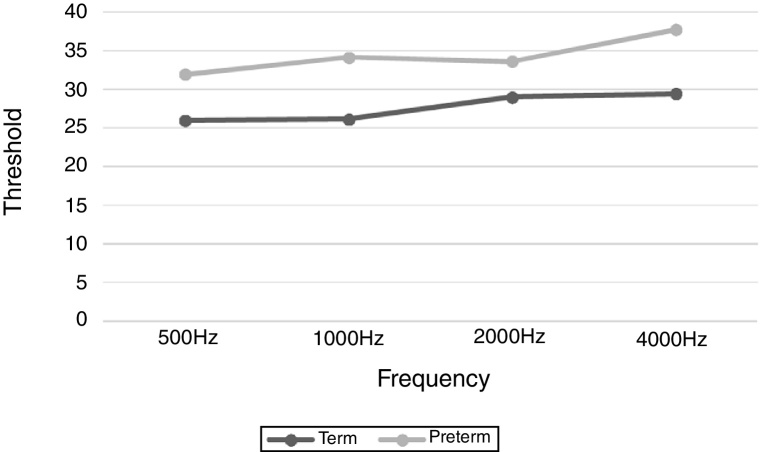
Minimum level of cortical auditory response between groups.

Regarding the threshold levels of cortical auditory response, there was a statistically significant difference between the groups for most of the frequencies evaluated, with higher values for the preterm group (Table 4 and Fig. 2). There was no statistically significant difference between ear and sex (*p* > 0.05). Analysis of variance (ANOVA) did not detect differences in the frequencies evaluated in both groups (*p* > 0.05).

**Table 4:** Comparison of threshold level of cortical auditory response for the different frequencies tested between groups.

Frequency	Group	Average ± SD	Median	1°Q	2°Q	3°Q	Minimum	Maximum	*p*-value
500 Hz	Term (*n* = 35)	26.00 ± 8.81	30.00	15.00	30.00	30.00	5.00	40.00	0.04^a^
	Preterm (*n* = 23)	31.96 ± 10.41	30.00	25.00	30.00	35.00	15.00	60.00	
1000 Hz	Term (*n* = 35)	26.14 ± 6.97	30.00	25.00	30.00	30.00	0.00	35.00	0.00^a^
	Preterm (*n* = 23)	34.13 ± 11.34	30.00	30.00	30.00	40.00	15.00	60.00	
2000 Hz	Term (*n* = 35)	29.00 ± 7.65	30.00	30.00	30.00	35.00	0.00	40.00	0.08^a^
	Preterm (*n* = 22)	33.64 ± 11.03	30.00	30.00	30.00	40.00	5.00	60.00	
4000 Hz	Term (*n* = 35)	29.43 ± 7.04	30.00	25.00	30.00	30.00	15.00	50.00	0.05^a^
	Preterm (*n* = 22)	37.73 ± 11.92	30.00	30.00	30.00	35.00	25.00	65.00	

SD, Standard Deviation; Q, Quartile.

The significance level of 5% was adopted.

a *U*-Mann–Whitney test.

Table 5 shows the percentage of detectable threshold responses by stimulus intensity in both groups.

**Table 5:** Percentage of the presence of a detectable threshold level CAEP in different intensities in both groups.

	Term				Preterm			
	500 Hz	1000 Hz	2000 Hz	4000 Hz	500 Hz	1000 Hz	2000 Hz	4000 Hz
30 dBHL	77%	97%	71%	74%	60%	60%	50%	59%
35 dBHL	94%	100%	94%	94%	75%	70%	75%	75%
40 dBHL	100%	100%	100%	97%	91%	88%	88%	84%
45 dBHL	100%	100%	100%	97%	91%	88%	88%	84%
50 dBHL	100%	100%	100%	100%	95%	91%	95%	84%
55 dBHL	100%	100%	100%	100%	95%	91%	95%	95%
60 dBHL	100%	100%	100%	100%	100%	100%	100%	95%
65 dBHL	100%	100%	100%	100%	100%	100%	100%	100%

**Figure 3:**
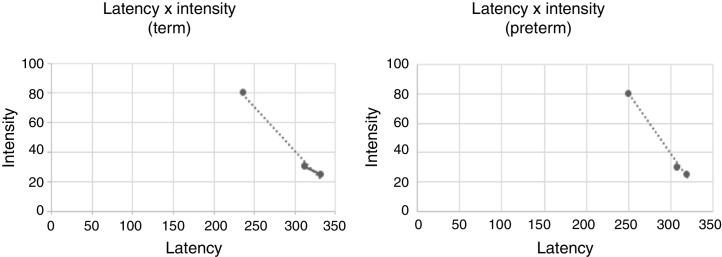
Graph of the latency versus intensity in both groups.

Figure 3 shows a graph of the latency and intensity function in both groups.

## Discussion

Electrophysiological assessments are fundamental for audiologic diagnosis in children younger than 6 months. The evaluation of brainstem auditory evoked potentials and stable auditory evoked potentials is routine in clinical practice and well documented in the scientific literature. On the other hand, the use of CAEP has been reported in older children and adults as an auxiliary method in audiological diagnosis, especially in cases where it is not possible to obtain consistent responses in short-latency auditory evoked potentials such as auditory neuropathy and in cases of neurological changes, but there is a lack of information in very young children, as in neonates.

In this study, it was possible to obtain cortical auditory response thresholds in term and preterm newborns with a positive result in neonatal hearing screening, evaluated at the same gestational age. The mean values were 26; 26.14; 29 and 29.43 dBHL for the full-term and 31.96; 34.13; 33.64 and 37.73 dBHL for the preterm neonates, for the frequencies of 500, 1000, 2000 and 4000 Hz, respectively. Comparison of these values with a study performed in the same outpatient clinic evaluating adults with normal hearing^2^ with the Hearlab System demonstrated differences in the minimum levels of responses between the neonates of the present study and the adults in the study cited. Researchers^2^ described mean values of 18.23 dBHL for 500 Hz, 15.9 dBHL for 1000 Hz, 15.97 dBHL for 2000 Hz and 17 dBHL for 4000 Hz. These differences in neonates of both term and preterm subjects of present study with the adults of the study cited^2^ support the contention that maturation influences cortical potentials, with each age group having responses characteristics as a function of the maturational process.^7^, ^8^

Comparing the latency and amplitude of the response at 80 dBHL between groups, there was a statistically significant difference in the latency of 4000 Hz, being higher for the preterm group. Studies^32^, ^33^ show that central auditory maturation can be impaired with preterm birth, and others^34^, ^35^ point to the fact that alterations in cortical auditory evoked potentials are suggestive and predictive of language and cognitive alterations throughout child development. The central auditory areas related to the higher frequencies are the first to mature^36^ which may explain the difference found in 4000 Hz.

In comparisons between groups, statistically significant differences were found for most frequencies (500, 1000 and 4000 Hz) when the threshold level of cortical response was evaluated, being higher for the preterm group. These results are expected due to maturational differences between groups. A meta-analysis study^33^ showed differences in the development of the auditory pathways of full-term and preterm neonates at the brainstem level. Thus, it is suggested that this immaturity extends to the central level, which was reflected in higher threshold levels of cortical responses in this group, even considering the corrected age.

Higher CAEP thresholds of the present study were observed compared with other studies involving brainstem auditory evoked potentials and steady-state auditory evoked responses in neonates. For the full-term group, the mean values were 26; 26.14; 29 and 29.43 dBHL. Researchers^37^ evaluated 30 full-term newborns by steady-state auditory evoked responses and obtained averages of 25.5; 17.8; 15.3 and 16.3 dBHL for 500, 1000, 2000 and 4000 Hz. In another study with brainstem auditory evoked potentials^38^ the electrophysiological thresholds varied from 0 to 20 dBHL for tone burst stimuli of 500 to 4000 Hz. A meta-analysis^39^ reported electrophysiological thresholds for brainstem auditory evoked potentials in children with normal hearing to have a mean of 19.5; 17.4; 13.6 and 15.5 dBHL for 500, 1000, 2000 and 4000 Hz, reflecting the faster maturation of the brainstem structures compared to the central structures.

In the present study, it was found that the ability to detect the cortical response P1i increased as the intensity of the stimulus increased in both groups. These results are in agreement with another study^40^ where the authors looked for the presence of the P1 component of children between 4 and 12 months of age; it was identifiable in 77% at an intensity of 30 dBSPL and 96% at 60 dBSPL. In the present study the response range was 0–50 dBHL for full-term infants and 5–65 dBHL for preterm infants. Other investigators^18^ also described similar results, with variation of 0–50 dBHL to identify the P1 potential in term newborns, which shows that the minimum threshold responses may be higher in this population due to the maturational process.^4^

In this study, we observed an increase in latency with a decrease in intensity. These results corroborate a similar study^18^ reporting that, using other electrophysiological assessments, the intensity of the acoustic stimulus influenced the latency of the responses due to reduced neural stimulation.^4^ This finding is important to assist the clinician in performing the examination and identification of the P1 component.

From the results described, it can be seen that the maturation of the central nervous system directly influences the minimum thresholds of cortical response. Obtaining the minimum levels of responses in this population is possible, but requires caution in interpretation due to neurological immaturity, especially in preterm births, since there is a great variation in dBHL of the level of response found, perhaps due to variability of the gestational age in this study. It is believed that CAEP may be used to supplement the audiological diagnosis, especially in populations with neurological alterations, in which reliable behavioral responses can not be obtained and short-latency auditory evoked potentials may present inconclusive results due to altered neural synchrony in patients.

Therefore, it is necessary to study the different age groups in the child population in order to better understand the differences in the threshold levels of cortical auditory response as a function of auditory maturation and, in addition, to establish criteria for this evaluation. The automatic analysis device contributes to a greater reliability of results obtained and may allow the clinician to rethink the use of these potentials in research and in the future, in clinical practice, striving for greater reliability in the audiological diagnosis and consequently quality of life in cases of neurological alterations.

## Conclusion

It was possible to obtain latency and amplitude values at 80 dBnNA and a threshold value for cortical responses in term and preterm newborns, with different results between groups, with higher values for preterm births. Thus, the use of CAEP to obtain minimum levels of responses in neonates should be cautious, always correlating with the audiological assessments already established in the scientific literature.

## Conflicts of interest

The authors declare no conflicts of interest.
